# A Study on the Dynamic Tunning Range of CVD Graphene at Microwave Frequency: Determination, Prediction and Application

**DOI:** 10.3390/nano12244424

**Published:** 2022-12-11

**Authors:** Hao Chen, Zhen-Guo Liu, Ming-Yang Geng, Xiang-Yu Meng, Wan-Lin Fu, Lu Ju, Bu-Yun Yu, Wu Yang, Yun-Qian Dai, Wei-Bing Lu

**Affiliations:** 1 State Key Laboratory of Millimeter Waves, School of Information Science and Engineering, Southeast University, Nanjing 210096, China; 2 Center for Flexible RF Technology, Frontiers Science Center for Mobile Information Communication and Security, Southeast University, Nanjing 210096, China; 3 Purple Mountain Laboratories, Nanjing 211111, China; 4 School of Chemistry and Chemical Engineering, Southeast University, Nanjing 210096, China

**Keywords:** graphene, CVD, tunning range, mathematic model, waveguide method

## Abstract

In recent years, graphene has shown great application prospects in tunable microwave devices due to its tunable conductivity. However, the electromagnetic (EM) properties of graphene, especially the dynamic tunning characteristics, are largely dependent on experimental results, and thus are unable to be effectively predicted according to growth parameters, which causes great difficulties in the design of graphene-based tunable microwave devices. In this work, we systematically explored the impact of chemical vapor deposition (CVD) parameters on the dynamic tunning range of graphene. Firstly, through improving the existing waveguide method, the dynamic tunning range of graphene can be measured more accurately. Secondly, a direct mathematical model between growth parameters and the tunning range of graphene is established. Through this, one can easily obtain needed growth parameters for the desired tunning range of graphene. As a verification, a frequency tunable absorber prototype is designed and tested. The good agreement between simulation and experimental results shows the reliability of our mathematic model in the rapid design of graphene-based tunable microwave devices.

## 1. Introduction

Carbon-based materials are an important member of the material family, and their inherent excellent physical or chemical properties have attracted extensive attention in various disciplines and fields. Graphene, a planar monolayer of carbon atoms arranged in honeycomb structures, has recently sparked intense and multidisciplinary research since the advent of free-standing graphene in 2004 [1]. In terms of electromagnetic (EM) field, graphene provides a new perspective to realize active surfaces because the charge density on graphene can be electrically controlled by applying a DC voltage, which makes it outstanding in research on dynamic tunable devices, such as switches [2], regulators [3,4,5], plasma [6,7], stealth [8,9], beam steering [10,11,12,13,14,15], and absorbers [16,17,18,19]. Therefore, graphene has rapidly become a hot spot in the fields of materials, energy, and information technology.

The early research on graphene was mostly theoretical simulation, and its EM modeling was based on Kubo’s formula [20]. During the design process, the researchers more or less ignored the feasibility of the experimental realization. Owing to the progress in manufacturing high-quality and large-scale graphene, microwave active devices based on graphene have been studied widely and constantly with experimental breakthroughs. In 2010, roll-to-roll production of 30-inch graphene films using the chemical vapor deposition (CVD) approach was reported [21]. In 2015, the work from Bilkent University successfully modulated the conductivity of graphene at the decimeter scale using graphene sandwich structure (GSS) [22]. The emergence of these key technologies further prompted researchers to gradually shift the focus of graphene research from pure theoretical design to the combination of theory and experiment. In particular, microwave and millimeter-wave devices based on graphene have made certain progress in the world due to practical application requirements [14,15,16,17,18,19,23,24].

In the microwave range, graphene can be considered a frequency-independent resistive film [15], and its tunable sheet resistance can be controlled by applying bias voltage through GSS. This characteristic has been widely applied to the modeling and design of graphene-based microwave tunable devices, such as wavefront controllers [15], absorbers, [18,19], attenuators [25], etc. The tunning range of graphene not only provides the basis for the simulation and design of these works but also serves as an important basis for experimental feasibility, but the tunning range of graphene in these works shows apparent differences, which causes doubts in researchers’ minds and is not conducive to the further application of graphene in microwave devices. In our previous work [26], the relationship between growth parameters and static sheet resistance of graphene was simply introduced. Many researchers are also committed to the study of the synthesis and also the physicochemical properties of graphene [27,28]. Raman, SEM and statistical techniques are applied to characterize the properties of graphene, such as the number of layers, growth uniformity, and so on. The microwave tunning property of graphene is largely dependent on its synthesis conditions, namely, the growth parameters for CVD process. However, the relationship between the dynamic tunning range of graphene and the growth parameters, which is of great importance to improve the design efficiency of graphene-related tunable devices, has never been studied in previously reported works. The main difficulty faced by the study of the relationship between the graphene tunning range and the growth parameters is twofold. Firstly, unlike the static sheet resistance of graphene, which can easily be obtained by the four-probe method, the dynamic sheet resistance of graphene cannot be accurately measured by existing methods because of the essential sandwich structure. Secondly, the CVD method is a sophisticated and time-consuming process, which brings trouble to either data collection or data processing.

To address these problems, in this work, firstly, with the help of transmission line theory, the measuring method of GSS is improved to help us obtain more precise tunning ranges of graphene. Secondly, the influence of growth parameters on the dynamic tunning property of graphene is investigated both qualitatively and quantitatively. To better reflect the numerical relation between the tunning and growth parameters, several statistical methods, including orthogonal experiment design [29,30] and multivariate nonlinear fitting [31,32], are adopted, based on which quantitative relations are given to easily obtain the desired tunable characteristic of graphene. At the end of this work, a tunable microwave absorber prototype is designed and used to verify the accuracy and availability of our model. The method adopted in this work is good guidance for following graphene-related works and has positive significance for promoting the combination of basic research and application research of two-dimensional (2D) materials.

## 2. Materials and Methods

Copper (25 μm) and CVD furnace (OTF 1200-X) were purchased from Hefei Kejing Material Technology Co. Ltd., Hefei, China, Hydrochloric acid (HCl, 36%) and nitric acid (HNO3, 70%) were purchased from Nanjing Wanqing Co. Ltd., Nanjing, China, Hydrogen (H2, 99.999%) and Methane (CH4, 99.999%) were purchased from Nanjing Shangyuan Co. Ltd., Nanjing, China, Polyvinyl chloride (PVC, 70 μm) was purchased from Shanghai Lingmin Trading Co. Ltd., Shanghai, China, Diaphragm paper (NKKTF4030) was purchased from Guangdong Canrd New Energy Technology Co. Ltd., Guangzhou, China.

Ionic liquid (methoxyethyldiethylmethylammomium bis((trifluoromethyl)sulfonyl)-imide, C_10_H_20_F_6_N_2_O_5_S_2_, CAS: 464927-84-2) was purchased from Lanzhou Institute of chemical physics. Wire mesh (125 μm, 0.5 Ω/sq) and Polydimethylsiloxane (PDMS, 1 mm) are purchased from CS New Materials Co., Ltd., Jining, China.

**Figure 1:**
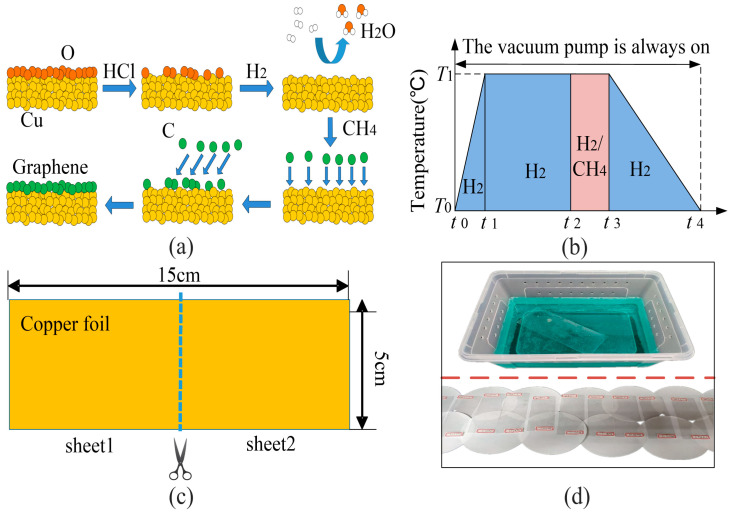
(**a**) Schematic diagram of CVD on copper foil. (**b**) Growth curve of graphene. (**c**) Schematic diagram of copper foil used to grow graphene. (**d**) Typical photographs during the synthesis of graphene. Upper: etching the copper foil in acid. Lower: obtained graphene samples on PVC substrate.

Large-area graphene is synthesized by CVD on copper foil. Copper foil is placed on a quartz holder in a CVD furnace. The mechanism of CVD on copper foil and the growth curve are shown in Figure 1a,b. After terminating the growth by stopping the flow of methane, the samples were cooled down to room temperature. Then we laminated 70-μm-thick PVC sheets on graphene-coated copper foils. Following the lamination, the copper foils were etched in diluted nitric acid solution and dried overnight to reduce the chemical doping of nitric acid on graphene. In order to meet the needs of subsequent measurements, the schematic diagram of the copper foil used to synthesize graphene is shown in Figure 1c. The size of each copper foil is 50 × 150 mm^2^, which can be cut into two pieces, subsequently, the two pieces can be combined into a GSS, typical photographs of synthesized graphene are shown in Figure 1d.

**Figure 2:**
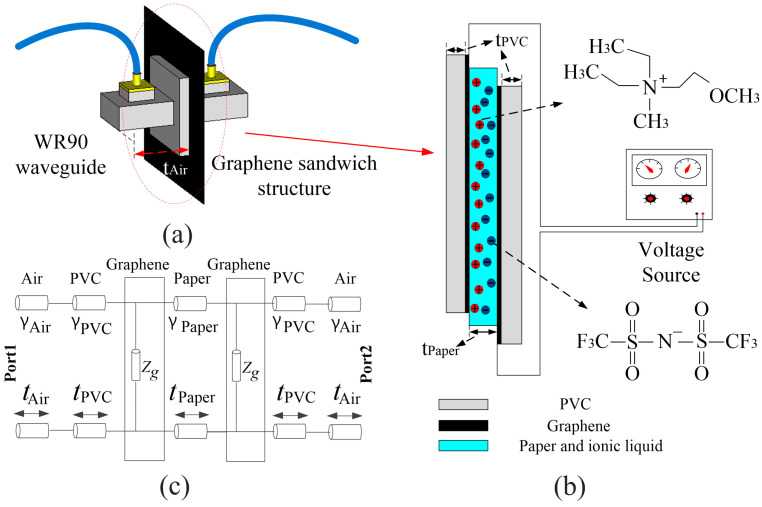
(**a**) Schematic diagram of the waveguide method. (**b**) Schematic diagram of GSS used to regulate graphene square resistance. (**c**) The equivalent circuit of the waveguide method.

As shown in Figure 2a,b, the GSS structure made of PVC, graphene, and ionic liquid is used to realize dynamic control of graphene sheet resistance [15,18,19,22]. PVC acts as the transfer carrier of graphene. The diaphragm paper is used as the carrier of ionic liquid, then the positive and negative electrodes are applied to upper graphene and lower graphene respectively. The ions of the electrolyte (ionic liquid) have very low mobility; therefore, they cannot respond to the electric field of microwaves. When the electrostatic field bias is applied, the electrolyte layer polarizes and ionic double layers form on the graphene–electrolyte interface with opposite polarizations, then the sheet resistance of GSS can be tuned due to the electrostatic doping on graphene electrodes [22].

During the measurement, each GSS is measured four times for reducing test error by changing the relative position between GSS and waveguide.

The absorption rate of the graphene-based absorber was tested by the waveguide method, and the waveguide used is WR62 (11.9–18 GHz). The absorption rate *A* is calculated through

(1) $$A=1-R-T=1-|S_{11}{|}^{2}-|S_{21}{|}^{2},$$

where *S*_11_ and *S*_21_ are reflection and transmission coefficients. Due to the metallic ground structure of designed absorber, the transmission is blocked. As such, *S*_21_ equals zero, so *A* is calculated through only *S*_11_ parameters.

## 3. Results

### 3.1. Improvement of Waveguide Method

The dynamic tunning range of graphene is measured through the waveguide method, as shown in Figure 2a, which is a kind of noncontact measurement method. It should be noted that the dynamic tunning range is unable to be measured by the commonly used four-probe method [17,26] because the conductive part (graphene) is wrapped by its transfer substrate. The waveguide used for measuring the dynamic sheet resistance of graphene is WR90 (8.2–12.4 GHz) and the vector network analyzer used is purchased from Agilent Co. Ltd., Santa Clara, CA, USA, (ZNB-40, 10–40 GHz).

To accurately obtain the sheet resistance of graphene, we improved the calculation process of the previously reported waveguide method [33,34] with the help of transmission line theory. The equivalent circuit of the waveguide method is shown in Figure 2c. Under the main mode (TE10) of the waveguide in our study (WR90), the complex propagation constant *γ* and characteristic impedance *Z* of the medium (air, PVC, paper) in the cross-section of the waveguide can be expressed by the following Formulas (2)–(4):

(2) γ(ω)=jβ(ω),

(3) $$\beta (\omega )=\sqrt{{\left(\frac{\omega }{c}\right)}^{2}\varepsilon_{r}-{\left(\frac{\pi }{a}\right)}^{2}},$$

(4) $$Z(\omega )=\frac{\omega \mu_{0}}{\beta (\omega )}.$$

in which c is the speed of light, *ε_r_* is the relative permittivity of the waveguide-filled medium, *μ*_0_ is the free space permeability, *β*(*ω*) is the phase constant, and *t* is the thickness of various mediums along the waveguide direction. The thickness of air part (35 mm) can be found in the datasheet of WR90 waveguide. The thicknesses of PVC and paper are 70 μm and 50 μm, respectively, and the relative permittivity of air, PVC, and paper are 1, 3, and 2.5, respectively.

The different transfer matrices are connected in cascade to obtain the response of the entire waveguide section as shown in Formulas (5)–(7):

(5) $$T_{Total}=T_{Air}T_{\mathrm{PVC}}T_{Graphene}T_{Paper}T_{Graphene}T_{\mathrm{PVC}}T_{Air}.$$

where

(6) $$T_{Air,\mathrm{PVC},Paper}=\left(\begin{array}{cc} \mathrm{cos h}(\gamma t) & Z\mathrm{sin h}(\gamma t)\\ \mathrm{sin h}(\gamma t)/Z & \mathrm{cos h}(\gamma t) \end{array}\right)$$

The total transmission matrix *T_T_*_otal_ can be obtained from the measured scattering parameters [35]:

(7) $$T_{total}=\left(\begin{array}{cc} {((1+S}_{11}{)(1-S}_{22}{)+S}_{12}S_{21}{)/(2S}_{21}) & Z_{\mathrm{Air}}{((1+S}_{11}{)(1+S}_{22}{)-S}_{12}S_{21}{)/(2S}_{21})\\ {((1-S}_{11}{)(1-S}_{22}{)-S}_{12}S_{21}{)/(2S}_{21}Z_{\mathrm{Air}}) & {((1-S}_{11}{)(1+S}_{22}{)+S}_{12}S_{21}{)/(2S}_{21}) \end{array}\right).$$

After obtaining *T_total_*, we can derive the following relationship:

(8) $$T_{Graphene}T_{Paper}T_{Graphene}=T_{\mathrm{PVC}}^{-1}T_{Air}^{-1}T_{Total}T_{Air}^{-1}T_{\mathrm{PVC}}^{-1}.$$

Traditionally, the influence of diaphragm paper and PVC is neglected [33,36]. Under such conditions, (8) can be simplified as

(9) $$T_{Graphene}T_{Graphene}=T_{Air}^{-1}T_{Total}T_{Air}^{-1}.$$

The sheet resistance of graphene can be easily calculated; however, it also brings certain errors, which is not conducive to future calculations using other types of graphene transfer carriers and ionic liquid carriers.

Here, we do not ignore the influence of PVC and paper and use the definition of matrix calculation to solve the formula. Assuming that

(10) $$T_{Graphene}=\left(\begin{array}{cc} T_{1} & T_{2}\\ T_{3} & T_{4} \end{array}\right),T_{Paper}=\left(\begin{array}{cc} T_{01} & T_{02}\\ T_{03} & T_{04} \end{array}\right),T_{\mathrm{PVC}}^{-1}T_{Air}^{-1}T_{Total}T_{Air}^{-1}T_{\mathrm{PVC}}^{-1}=\left(\begin{array}{cc} T_{05} & T_{06}\\ T_{07} & T_{08} \end{array}\right),$$

From the previous description, it can be seen that *T*_01_–*T*_08_ are all known values, while *T*_1_–*T*_4_ are the unknowns to be solved. According to (8), we can obtain the values of *T*_1_–*T*_4_ by listing and solving the following Equation (11):

(11) $$\left\{\begin{array}{c} T_{1}(T_{1}T_{01}+T_{2}T_{03})+T_{3}(T_{1}T_{02}+T_{2}T_{04})=T_{05}\\ T_{2}(T_{1}T_{01}+T_{2}T_{03})+T_{4}(T_{1}T_{02}+T_{2}T_{04})=T_{06}\\ T_{1}(T_{3}T_{01}+T_{4}T_{03})+T_{3}(T_{3}T_{02}+T_{4}T_{04})=T_{07}\\ T_{2}(T_{3}T_{01}+T_{4}T_{03})+T_{4}(T_{3}T_{02}+T_{4}T_{04})=T_{08} \end{array}\right.$$

It should be noted that there are four groups of roots of the equation, which need to be decided by judgment. Theoretically, according to the form of the transmission matrix of graphene, (*T*_1_, *T*_2_; *T*_3_, *T*_4_) should satisfy the form of (1, 0; 1/*R*_S_, 1). Thereout, we can select the solution with practical physical significance, so that the sheet resistance of graphene can be obtained by *R*_S_ = 1/*T*_3_.

**Figure 3:**
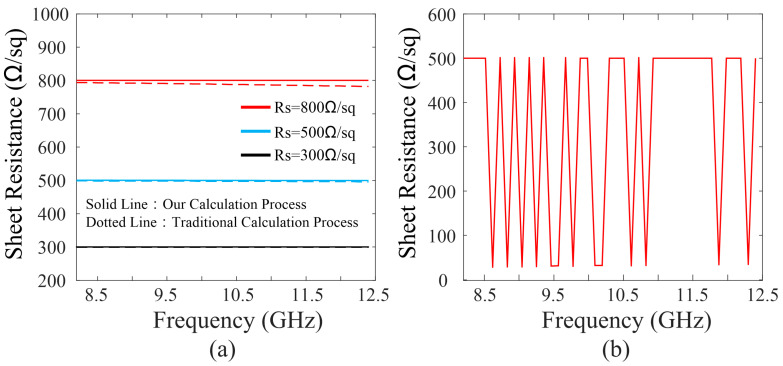
(**a**) Derived sheet resistance corresponding to *R*_S_ = 300, 500, and 800 Ω/sq using the traditional method and our improved method. (**b**) Derived sheet resistance corresponding to *R*_S_ = 500 Ω/sq without judgment procedure.

As a verification of the improved method, we simulated the S parameters corresponding to *R*s = 300, 500, and 800 Ω/sq and substitute them into (7), then the retrieved sheet resistance can be obtained through (9) or (11). In Figure 3a, the sheet resistance can be correctly calculated by our improved method, in contrast, appear deviations could be observed while using the traditional method, especially with the increase in frequency. For completeness, the retrieved result corresponding to *R*_S_ = 500 Ω/sq without judgment procedure is plotted in Figure 3b, the sharp jumps of the result are caused by the multi-foot of the Equation (11), which implies the necessity of the judgment procedure.

The improved method is important for the measurement and characterization of the sandwich structure, mathematically, the reason lies in that the matrix equation of **A** × **B** × **A** = **C** cannot be solved directly through matrix transformation, in which, **A**, **B**, and **C** are 2 × 2 matrices. This improved method is used for the measurement of dynamic tunning range of GSS in this work, it also can be applied to other materials, such as planner or powder materials.

### 3.2. Impact of Growth Parameters

The mechanism of CVD on the copper foil can be understood with the help of a schematic diagram and growth curve shown in Figure 1a,b. As shown in Figure 1a, for metals such as copper having a low carbon dissolution rate, the graphene is formed with the carbon atoms depositing to the copper foil through a surface growth mechanism. Both the aim of foil pretreatment by hydrochloric acid and annealing step by hydrogen are to remove the surface oxides and other impurities of copper foil. The annealing step also increases the flatness of copper foil. Methane is used as the carbon source, which cracks at high temperature, the obtained carbon atoms will be adsorbed on the surface of copper, then the atoms nucleate and grow into graphene islands. With the constant expanding of these islands, they connect each other, thus obtaining graphene pieces.

From above, the quality of graphene the factors affecting the quality of graphene mainly include surface cleanliness, surface flatness of copper, and the amount of carbon atoms originating from methane. Combined with the growth curve shown Figure 1b, four parameters are selected for study: *T*_1_, Δ*t*_1_, Δ*t*_2_, and *Ratio*. In which, *T*_1_ is annealing temperature, Δ*t*_1_ = *t*_2_ − *t*_1_ is the annealing duration, Δ*t*_2_ = *t*_3_ − *t*_2_ is growth duration, *Ratio* is the flow ratio of hydrogen to methane during the growth stage.

**Figure 4:**
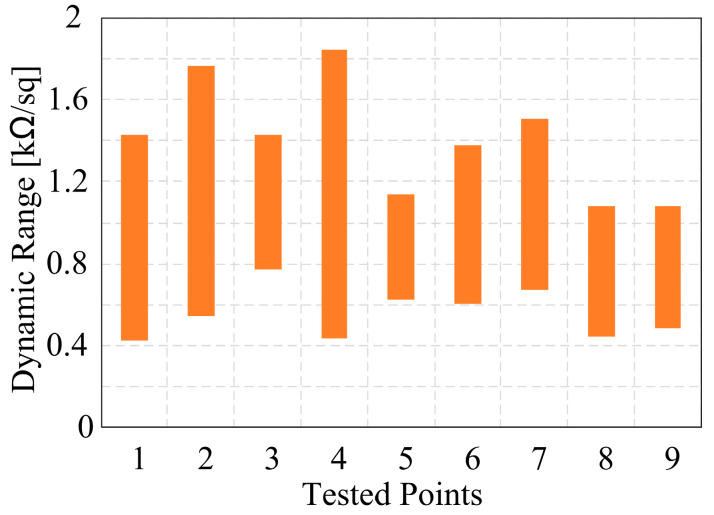
Calculated dynamic range of graphene samples in orthogonal experiment.

**Figure 5:**
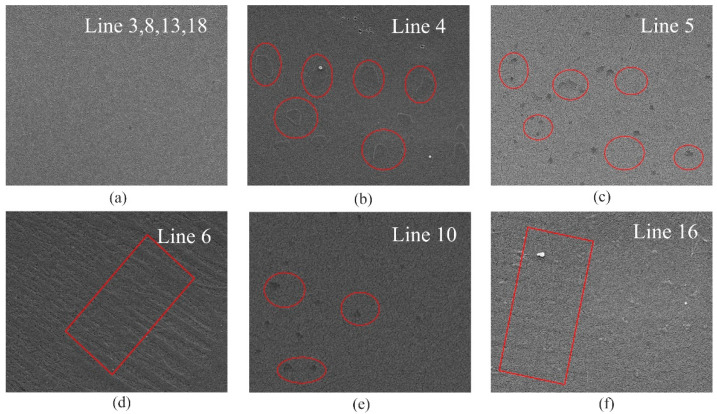
Typical SEM images of samples corresponding to line (**a**) 3, 8, 13, 18, (**b**) 4, (**c**) 5, (**d**) 6, (**e**) 10, and (**f**) 16 in Table 2. The scale of the pictures was 3 μm.

To investigate the influence of each parameter, the specific parameter should fluctuate around an initial value while keeping the other three parameters unchanged (single variable principle). The first problem to be solved is the initial values of *T*_1_, Δ*t*_1_, Δ*t*_2_, *Ratio*. Physically, the graphene synthesized under this group of values has the largest dynamic range, and an orthogonal experiment [29,30] is used to reduce the time consumption while ensuring the accuracy of experimental results as much as possible. The orthogonal table generated by SPSS software (v17.0) is shown in Table 1. Where the variation ranges of *T*_1_, Δ*t*_1_, Δ*t*_2_, *Ratio* are [1000, 1050] (°C), [60, 180] (mins), [10, 30] (mins), [70:30, 50:50] (sccms), respectively. The measured results of the orthogonal experiment is shown in Figure 4, the tunning range of GSS composed of graphene sheets measured by waveguide method in group_4 is the largest, which varies from 430−1840 Ω/sq. Combining our previous work [26], it can be seen that the demands for growth parameters are consistent when pursuing the lowest static sheet resistance and the biggest tunning range of graphene. This can be understood by the best flatness and integrity of graphene sample grown under the parameter of group_4 as shown in Figure 5a. Based on which, (*T*_1_, Δ*t*_1_, Δ*t*_2_, *Ratio*) = (1025, 180, 20, 70:30) is used as the initial value of the parameter comparison experiment.

**Figure 6:**
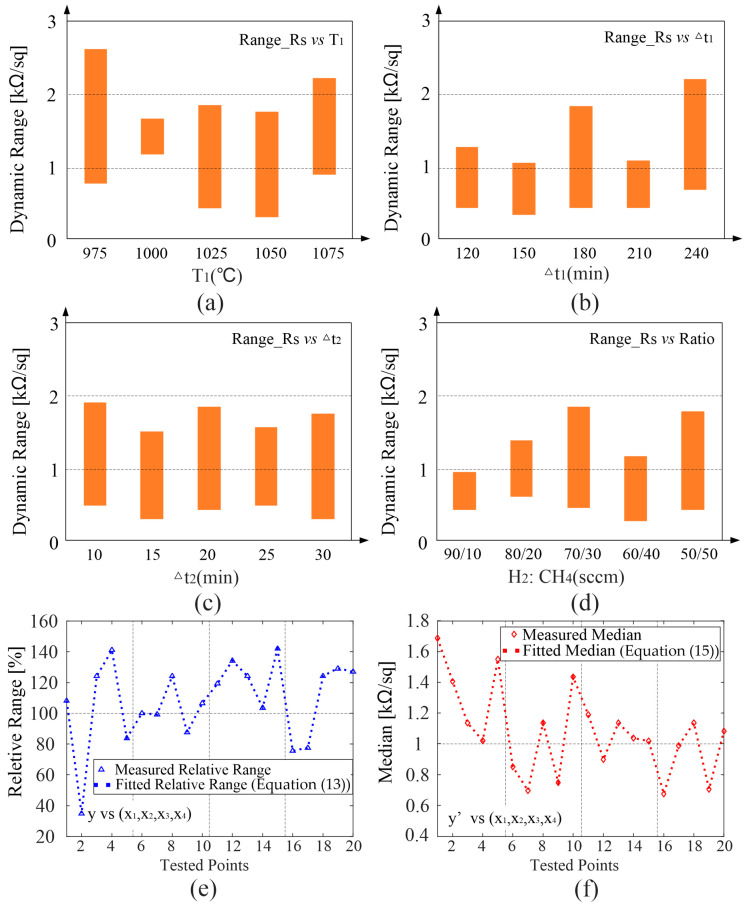
Calculated (**a**–**d**) dynamic range of graphene samples shown in Table 2. (**e**) Fitting results between the relative dynamic range of graphene and all four factors. (**f**) Fitting results between the median of dynamic range and all four factors.

After obtaining the initial values of *T*_1_, Δ*t*_1_, Δ*t*_2_, *Ratio*, the second set of experiments, namely, parameter comparison, is carried out to explore the influence of a single parameter on the tunning range of graphene, as shown in Table 2. The results of parameter comparison experiments are shown in Figure 6. The relations between tunning range with annealing temperature *T*_1_, annealing duration Δ*t*_1_, growth duration Δ*t*_2_, and gas ratio are plotted in Figure 6a−d, respectively. From Figure 6a, although the lower annealing temperature *T*_1_ will lead to relatively low growth uniformity, it also ensures the upper limit of the dynamic tunning range of graphene. For example, when the annealing temperature is 975 °C, the upper and lower limits of the dynamic range of graphene are slightly higher than those of other groups. Graphene with lower static sheet resistance can be obtained by changing annealing duration Δ*t*_1_ or gas ratio, but as shown in Figure 6b,d, the upper limit of dynamic sheet resistance is also suppressed. For these three parameters, they influence on the annealing process of the CVD procedure, either the annealing temperature or annealing duration of copper have a significant effect on the recrystallization effect of copper foil, causing the typical problems of multilayer (Figure 5b), defects (Figure 5c) and out of flatness (Figure 5d) to the final graphene samples. While the ratio of hydrogen to methane especially the amount of hydrogen largely influences the reduction degree of oxides on the copper foil. From these, we can notice that the change of the variation range in Figure 6a,b,d is relatively severe because they are directly related to copper foil. These trends can be also understood from optical microscopy (OM) of copper foil [37]. From Figure 6c, we can see that not only the uniformity but also a wide range of dynamic range of graphene can be guaranteed by changing the growth duration Δ*t*_2_, the variation trend is relatively smooth. Together with the SEM pictures in Figure 5, it can be concluded that the flatness of graphene has the greatest influence on its tunning ability (Figure 5d,f), when obvious rolling marks are observed, the corresponding tunning ranges of graphene are suppressed, this may be caused by the uneven doping effect on graphene sheets. In contrast, a few defects originating from too high *T*_1_ or too long Δ*t*_1_ (Figure 5c,e) will increase the overall tunning range of graphene. This can be understood in that when patterns are made on graphene sheets, the effective conducting area decreases [38], leading to higher sheet resistance.

Here, we do not define what is “good” or “bad” about graphene. Readers can change the growth parameters of graphene according to the needs of practical applications to get the desired properties. The above qualitative analysis is useful for improving the quality of graphene. However, from the aspect of the application, especially in the field of electromagnetism, either single layer, multilayer, or graphene with some defects is modeled as a surface impedance boundary with its surface resistance. In the following, we quantitatively analyze the results of this section and give readers a more practical method to select growth parameters.

### 3.3. Mathematic Model

To effectively process the previous data and provide guidance for the subsequent work, we fitted the data and formed the mathematic model of the relationship between the dynamic tunning range and growth parameters of graphene. Different from the static sheet resistance, the dynamic range of graphene is not a single value, but an interval. To establish a quantitative model of the interval, one can choose to fit the relation among the upper, the lower bounds of the interval, and growth parameters. However, to better reflect the nature of the interval, we refer to the concept of relative bandwidth from the index of the absorber and define the concept of relative dynamic range:

(12) $$Reletive\_range=\frac{R_{s2}-R_{s1}}{(R_{s}{}_{1}+R_{s2})/2}=f(T_{1},\Delta t_{1},\Delta t_{2},Ratio)$$

in which *R*_S1_ and *R*_S2_ are the upper and lower bounds of the dynamic range. The statistical model is widely used in many fields, including the materials domain [39,40]. The specific tool is not unique, aiming at different kinds of problems. Here, in order to solve this kind of multivariable nonlinear fitting problem [31,32], the nlinfit tool in MATLAB is used [41,42], and the form of the model is as follows:

(13) $$y=\sum_{i=1}^{4}\sum_{j=1}^{4}q_{i,j}x_{i}{}^{5-j}{+q}_{0}.$$

where *y* represents the relative dynamic tunning range of graphene and *x*_1_–*x*_4_ the variation vector of annealing temperature, annealing duration, growth duration, and gas ratio, respectively. Polynomial fitting with fitting order equaling 4 is selected after comparing with other fitting functions. The values of q_i,j_ after optimization are shown in Table 3, and the value of q_0_ is 2.8767 × 107, the optimization process can be found in [26]. The physical significance of the model can be understood from the following aspects. Firstly, when the other three variables are fixed, and the three variables have been preliminarily optimized through orthogonal experiments, the effect of a single parameter (the fourth parameter) on the properties of GSS should be gradual rather than jump, thus smooth curve fitting (polynomial fitting is selected here) is used to preliminarily determine the variation trend of relative dynamic tunning range with the change of this parameter. Secondly, compared with individually fitting the variation trend of relative dynamic tunning range with the four parameters to form four equations:

(14) $$\left\{\begin{array}{c} y_{1}=f_{1}(x_{1})\\ y_{2}=f_{2}(x_{2})\\ y_{3}=f_{3}(x_{3})\\ y_{4}=f_{4}(x_{4}) \end{array}\right.$$

where *y*_1_–*y*_4_ denotes the change in graphene dynamic range with *x*_1_–*x*_4_. Equation (13) can be seen as a modified model, which not only can reflect the changing trend of graphene tunning range with a single variable but also includes the effect or other three parameters, thus increasing the reliability. As shown in Figure 6e, all measured results in the parameter comparison experiment can be correctly located on the fitted line. In addition, the model between the median of the tunning range and growth parameters of graphene is also determined using the same method:

(15) $$y^{\prime }=\frac{R_{s2}+R_{s1}}{2}=\sum_{i=1}^{4}\sum_{j=1}^{4}{q^{\prime }}_{i,j}x_{i}{}^{5-j}{+q^{\prime }}_{0}$$

Among them, *y′* represents the median of the tunning range of GSS. The values of q_i,j_ after optimization are shown in Table 4, and the value of q′_0_ is 3.7768 × 10^4^, and the corresponding fitting result is shown in Figure 6f.

After obtaining the mathematic models, one can not only directly predict the tunning range of graphene through the growth parameters but also obtain needed growth parameters for desired tunning range. Here, we make a simple demonstration of the second function of the model, namely to deduce the required growth parameters under the circumstance of the known desired tunning range of GSS.

When considering the desired range of tunable characteristics, both (13) and (15) are needed. For instance, the desired tunning range of graphene is 400 to 1400 Ω/sq, and we predetermine that *x*_1_ = 1025, *x*_3_ = 20, *x*_4_ = 70. The median of the range is 0.9 kΩ/sq, substituting it to (15), *x*_2_ = 119.2, 159.3, 201.1, and 231.8 can be obtained. Except for the out-of-range value 119.2, we calculate the corresponding relative dynamic range of graphene under the other three values of annealing duration through (13). The relative ranges are 112.34%, 101.86% and 81.27%, respectively, thus the tunable range of graphene in a GSS can be obtained as [394.5, 1405.5] Ω/sq, [441.6, 1358.4] Ω/sq, [534.2, 1265.5] Ω/sq. We can see that the desired range is involved in the interval of [394.5, 1405.5] Ω/sq, namely, the tunning range of around 400 to 1400 Ω/sq can be obtained using the parameter *x*_1_ = 1025, *x*_2_ =159.3, *x*_3_ = 20, *x*_4_ = 70, which will be verified by a frequency tunable absorber shown in Section 4.

It should be noted that when using the model to predict the dynamic tunning range of GSS or deduce the needed parameters based on desired tunning range, one should still obey the rule of single-parameter principle, namely, the other three parameters should equal the optimized value obtained by the orthogonal experiment. The reason lies in that the data used to calculate to coefficients of the model, i.e., the data of the parameter comparison experiment is originated from the initial value of (*T*_1_, Δ*t*_1_, Δ*t*_2_, *Ratio*) = (1025, 180, 20, 70:30), as stated in Section 3.

### 3.4. Application of the Model

**Figure 7:**
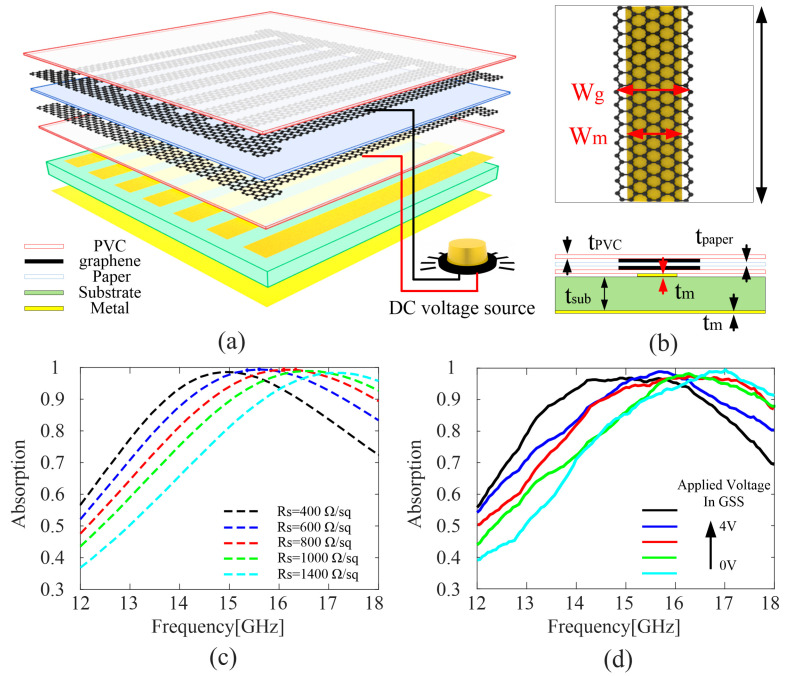
(**a**) Schematic of the absorber array. (**b**) Transverse view and longitudinal view of the unit cell. (**c**) Simulated and (**d**) measured results of the absorber.

In this section, a frequency tunable absorber is designed and fabricated to verify the effect of the mathematic model on the actual device. The overall structure of the absorber is shown in Figure 7a, which consists of a patterned GSS layer, metal ribbon layer, substrate layer, and bottom metal layer. The schematic diagram and corresponding size of the unit cell are shown in Figure 7b. The period of the unit is *p* = 2.7 mm, the width of the graphene ribbon is *w*_g_ = 2.2 mm, the side length of the metal ribbon is *w*_m_ = 1.6 mm. The substrate adopted here is PDMS, with relative permittivity of 2.8 and the thickness of *t*_sub_ = 1 mm. The thickness of the metal ribbon and bottom metal layer is *t*_m_ = 125 μm, and the sheet resistance is 0.5Ω/sq. Commercial software CST 2019 was used to simulate the model, in which unit-cell boundary condition is set up. The incident wave is vertical to the sample with the electric field perpendicular to the graphene strip. In the simulation, the sheet resistances of graphene ribbons are 400, 600, 800, 1000, and 1400 Ω/sq, respectively. As can be seen from Figure 7c, when the sheet resistance changes from 400 to 1400 Ω/sq, the central frequency of the absorption peak shifts from 14.9 to 17.0 GHz, while the absorption rate remains above 0.9, showing obvious frequency tunable absorption phenomenon.

**Figure 8:**
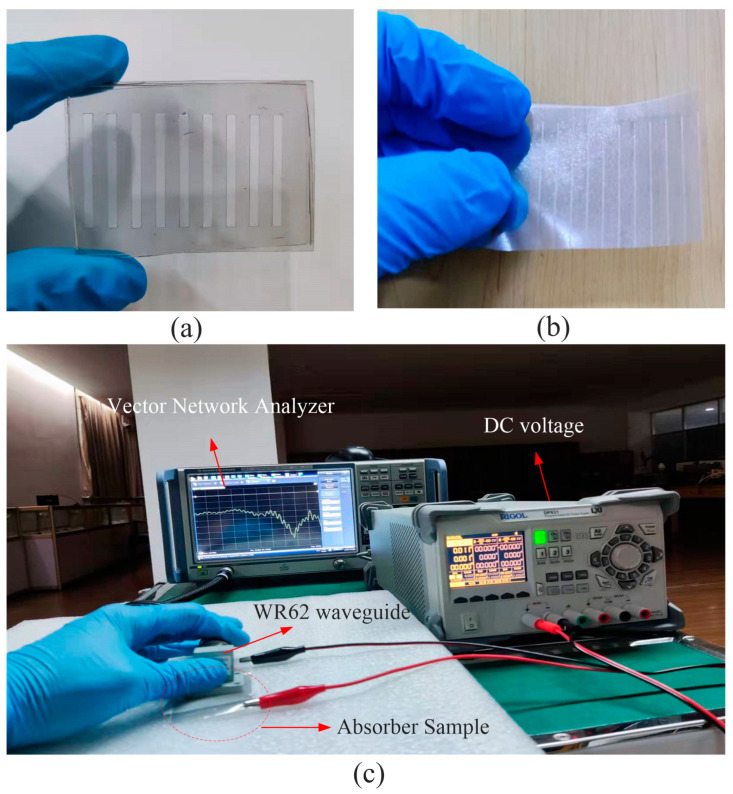
(**a**) Fabricated metal ribbon on PDMS substrate. (**b**) Fabricated patterned graphene. (**c**) Measurement environment of the absorber.

To verify the simulated results and also the correctness of the mathematical model, as calculated in the last section, the parameters *x*_1_ = 1025, *x*_2_ =159.3, *x*_3_ = 20, *x*_4_ = 70 are used to synthesize the graphene with a desired tunning range of 400 to 1400 Ω/sq. The graphene ribbon and metal ribbon are realized through a laser engraving machine, the material of the metal ribbon layer and the metal ground layer is transparent metallic wire mesh. The photograph of the metal ribbon on PDMS substrate and the patterned graphene is shown Figure 8a,b while the measurement environment is shown in Figure 8c. The test results are shown in Figure 7d, several results are recorded with the increase in applied voltage, it can be seen that the absorption peak shift from around 16.9 GHz to 14.9 GHz when the applied voltage increases from 0 to 4 V, which is basically consistent with the simulated results. The slight difference may be caused by the deviation in the tunning of graphene between simulation and measurement. In addition, the imperfect adhesion between the patterned GSS layer and the metal ribbon layer will also lead to certain deviations. In general, there is good agreement between the simulation and the actual measurement, which indicates the accuracy and useability of the fitting formula in this paper.

## 4. Conclusions

In summary, the influences of CVD parameters on the dynamic tunning range of graphene were systematically investigated. The measurement method of GSS is improved to obtain more accurate tunning ranges of graphene. Direct mathematical models between growth parameters and the tunability of graphene are given. As a result, one can quickly and precisely predict the tunning range of graphene through the growth parameters or obtain needed growth parameters for the desired tunning range, which largely improves the design efficiency of graphene-based microwave devices. At the end of the work, the usability and accuracy of the proposed mathematic relation are verified by a frequency tunable absorber. The results of this work have positive significance for promoting the research and application of novel materials.

## Figures and Tables

**Table 1:** Variation of parameters and results in the orthogonal experiment.

Sequence	*T*_1_ (°C)	Δ*t*_1_ (min)	Δ*t*_2_ (min)	*Ratio* (sccm)	Tunning Range (kΩ/sq)
1	1050	120	30	70:30	[0.43, 1.43]
2	1050	180	10	60:40	[0.54, 1.76]
3	1025	60	30	60:40	[0.77, 1.43]
4	1025	180	20	70:30	[0.43, 1.84]
5	1025	120	10	50:50	[0.62, 1.14]
6	1025	180	30	50:50	[0.60, 1.38]
7	1000	60	10	70:30	[0.67, 1.51]
8	1050	60	20	50:50	[0.44, 1.08]
9	1000	120	20	60:40	[0.49, 1.08]

**Table 2:** Variation of parameters and results in the contrast experiment.

Sequence	*T*_1_ (°C)	Δ*t*_1_ (min)	Δ*t*_2_ (min)	*Ratio* (sccm)	Tunning Range (kΩ/sq)
1	975	180	20	70:30	[0.78, 1.84]
2	1000	180	20	70:30	[1.16,1.65]
3	1025	180	20	70:30	[0.43, 1.84]
4	1050	180	20	70:30	[0.30, 1.74]
5	1075	180	20	70:30	[0.90, 2.20]
6	1025	120	20	70:30	[0.43, 1.28]
7	1025	150	20	70:30	[0.35, 1.04]
8	1025	180	20	70:30	[0.43, 1.84]
9	1025	210	20	70:30	[0.42, 1.08]
10	1025	240	20	70:30	[0.67, 2.20]
11	1025	180	10	70:30	[0.48, 1.90]
12	1025	180	15	70:30	[0.30, 1.50]
13	1025	180	20	70:30	[0.43, 1.84]
14	1025	180	25	70:30	[0.50, 1.58]
15	1025	180	30	70:30	[0.30, 1.74]
16	1025	180	20	90:10	[0.42, 0.93]
17	1025	180	20	80:20	[0.61, 1.37]
18	1025	180	20	70:30	[0.43, 1.84]
19	1025	180	20	60:40	[0.25, 1.16]
20	1025	180	20	50:50	[0.40, 1.77]

**Table 3:** Value of qi,j after optimization. (i = 1–4, j = 1–4).

	i	1	2	3	4
j					
1		2.4885 × 10^−5^	−0.1033	1.6068 × 10^2^	−1.1102 × 10^5^
2		1.0493 × 10^−5^	−0.0075	1.9464	−2.2040 × 10^2^
3		0.0037	−0.2364	5.1130	−42.2300
4		4.9760 × 10^−4^	−0.1350	13.4652	−5.8628 × 10^2^

**Table 4:** Value of q′_i,j_ after optimization. (i = 1–4, j = 1–4).

	i	1	2	3	4
j					
1		2.4885 × 10^−5^	−0.1033	1.6068 × 10^2^	−1.1102 × 10^5^
2		1.0493 × 10^−5^	−0.0075	1.9464	−2.2040 × 10^2^
3		0.0037	−0.2364	5.1130	−42.2300
4		4.9760 × 10^−4^	−0.1350	13.4652	−5.8628 × 10^2^

## Data Availability

Not applicable.
